# Long-lasting insecticidal net (LLIN) ownership, use and cost of implementation after a mass distribution campaign in Kasaï Occidental Province, Democratic Republic of Congo

**DOI:** 10.1186/s12936-016-1671-1

**Published:** 2017-01-09

**Authors:** Henry Maggi Ntuku, Laura Ruckstuhl, Jean-Emmanuel Julo-Réminiac, Solange E. Umesumbu, Alain Bokota, Antoinette Kitoto Tshefu, Christian Lengeler

**Affiliations:** 1 Kinshasa School of Public Health, Kinshasa, Democratic Republic of Congo; 2 Swiss Tropical and Public Health Institute, Basel, Switzerland; 3 University of Basel, Basel, Switzerland; 4 National Malaria Control Programme, Kinshasa, Democratic Republic of Congo

**Keywords:** Malaria, LLIN ownership, LLIN use, Mass distribution campaign, LLIN cost, Delivery strategy, Malaria prevalence, Democratic Republic of Congo

## Abstract

**Background:**

Long-lasting insecticidal nets (LLIN) are a highly effective means for preventing malaria infection and reducing associated morbidity and mortality. Mass free distribution campaigns have been shown to rapidly increase LLIN ownership and use. Around 3.5 million LLINs were distributed free of charge in the Kasaï Occidental Province in the Democratic Republic of Congo (DRC) in September–October 2014, using two different approaches, a fixed delivery strategy and a door-to-door strategy including hang-up activities.

**Methods:**

Repeated community-based cross-sectional surveys were conducted 2 months before and six months after the mass distribution. Descriptive statistics were used to measure changes in key malaria household indicators. LLIN ownership and use were compared between delivery strategies. Univariate and multivariate logistic regression analyses were used to identify factors associated with LLIN use before and after the mass distribution. A comparative financial cost analysis between the fixed delivery and door-to-door distribution strategies was carried out from the provider’s perspective.

**Results:**

Household ownership of at least one LLIN increased from 39.4% pre-campaign to 91.4% post-campaign and LLIN universal coverage, measured as the proportion of households with at least one LLIN for every two people increased from 4.1 to 41.1%. Population access to LLIN within the household increased from 22.2 to 80.7%, while overall LLIN use increased from 18.0 to 68.3%. Higher LLIN ownership was achieved with the fixed delivery strategy compared with the door-to-door (92.5% [95% CI 90.2–94.4%] versus 85.2% [95% CI 78.5–90.0%]), while distribution strategy did not have a significant impact on LLIN use (69.6% [95% CI 63.1–75.5%] versus 65.7% [95% CI 52.7–76.7%]). Malaria prevalence among children aged 6–59 months was 44.8% post-campaign. Living in a household with sufficient numbers of LLIN to cover all members was the strongest determinant of LLIN use. The total financial cost per LLIN distributed was 6.58 USD for the fixed distribution strategy and 6.61 USD for the door-to-door strategy.

**Conclusions:**

The mass distribution campaign was effective for rapidly increasing LLIN ownership and use. These gains need to be sustained for long-term reduction in malaria burden. The fixed delivery strategy achieved a higher LLIN coverage at lower delivery cost compared with the door-to-door strategy and seems to be a better distribution strategy in the context of the present study setting.

## Background

Long-lasting insecticidal nets (LLIN) are a highly effective means of preventing malaria infection and reducing associated morbidity and mortality, particularly in endemic areas [1, 2]. Across sub-Saharan Africa, the use of LLIN has been shown to be associated with an average parasite prevalence reduction of 20% [2]. Sustained high coverage of LLIN and other effective interventions is essential to achieve and maintain such gains in reduction of malaria burden, and therefore achieve the joint target of the new action and investment to defeat malaria (AIM) and the global technical strategy for malaria [3, 4]. Mass free distribution campaigns have been shown to rapidly increase LLIN ownership and use in several countries [5–7]. Across Africa, different distribution strategies such as fixed or door-to-door delivery have been used with varying effects on LLIN coverage and use. Furthermore, despite overall LLIN scale-up, several other factors still influence LLIN use including demographic characteristics; individual’s knowledge and beliefs related to malaria and LLIN; dwelling construction, family size, sleeping arrangements; LLIN characteristics; environmental factors; community and cultural characteristics; distribution strategy and household net density [5, 6, 8–11].

The Democratic Republic of Congo (DRC), through its National Malaria Control Programme (NMCP), is in the midst of unprecedented efforts to rapidly scale up coverage of malaria interventions. As recommended by the World Health Organization (WHO) to achieve universal coverage of LLIN, the NMCP has adopted a combined strategy of: free mass distribution campaigns every 3 years and routine distribution through antenatal care visits and immunisation services [12]. While the mass distribution has been shown to be the best approach to achieve rapid scale up (aiming to achieve at least 80% of people sleeping under a LLIN), routine distribution is important for maintaining high levels [13, 14].

Since the adoption of free of charge LLIN policy in 2006, over 75 million LLINs have been distributed across the country, leading to a tremendous increase in ownership and use [15]. For example, the overall proportion of households with at least one LLIN increased from 9% in 2007 to 70% in 2014 [16, 17]. However, the scale-up of these interventions has not been achieved across all geographic areas of the DRC. Results of the 2013–2014 Demographic and Health Survey (DHS) showed a strong coverage gradient between provinces with Orientale and Kasaï Occidental Provinces having the lowest ownership rate at 47 and 58%, respectively. Furthermore, the lowest LLIN use in children <5 years of age was reported in Kasaï Occidental at 36% [17].

Consequently, as part of a larger effort by many partners to accelerate the progress towards the goal of increasing coverage and use of LLIN, a mass distribution campaign was organized in 2014, distributing approximately 3,5 million LLINs in Kasaï Occidental using two different approaches, a fixed strategy and a door-to-door strategy with hang up activities. The aim of this research was to measure changes in key malaria household indicators before and after the LLIN mass distribution campaign, as well as malaria morbidity after mass distribution and to identify factors associated with LLIN use. This study also compared the two distribution strategies in terms of LLIN ownership, use and associated cost.

## Methods

### Study site

**Fig. 1:**
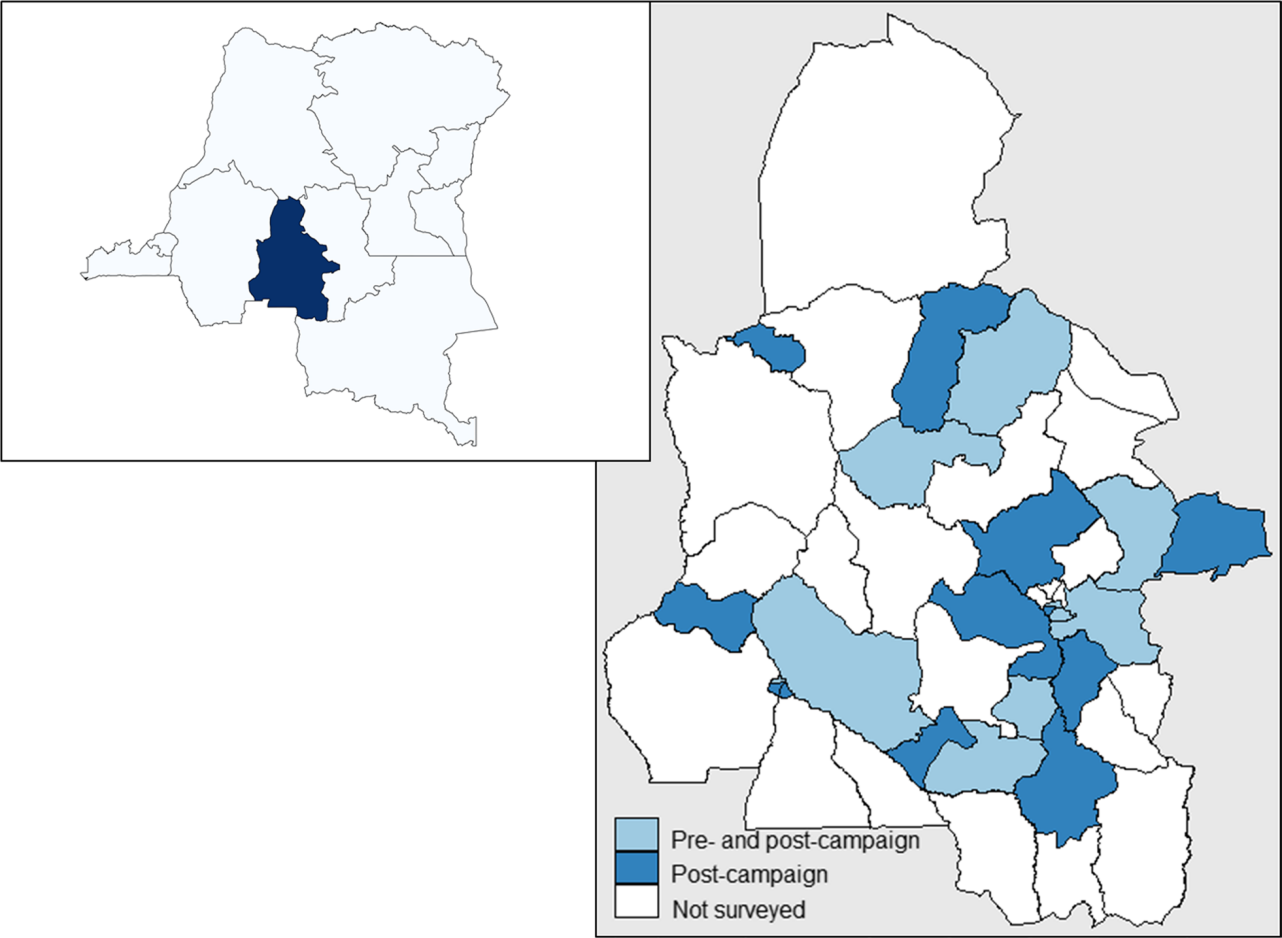
Map showing the location of the study sites

This study was conducted in the Kasaï Occidental Province, located in the centre of the Southern part of the DRC (Fig. 1). Kasaï Occidental spans over 170,000 km^2^ and has an estimated 7.3 million inhabitants. The province has two districts (Lulua and Kasaï) and one large city in each-Kananga and Tshikapa, respectively. On the health front, it is divided into 44 Health Zones (HZ) grouped into five Health Districts. The HZ represents the primary operational unit of the health system in DRC. It usually covers a population of 100,000–150,000 in rural areas and 200,000–250,000 in urban centres. It includes a general referral hospital, some health centres and about a dozen lower level health facilities. Each HZ is further divided into 15 health areas (HAs) on average, which represent the lowest level of the health system. Each HA is clearly delimited and defined by the Ministry of Health and usually has 10,000–15,000 inhabitants. In Kasaï Occidental Province, malaria is endemic with stable transmission throughout the year. The DHS 2014 reported an average malaria prevalence of 45% in children less than 5 years [17], one of the highest in the world. A previous mass distribution campaign in the province was organized in 2011.

### Mass distribution campaign

A free LLIN distribution campaign took place in all HZ of Kasaï Occidental Province in 2014 using two different strategies: (a) fixed delivery strategy; (b) door-to-door (hang up) strategy.

#### Fixed strategy

This strategy was used to distribute nets in 35 of the 44 HZ in Kasaï Occidental Province. Specially selected community volunteers were mobilized and trained to visit each household before the campaign. The volunteers registered the number of residents per household, issued a numbered coupon to be exchanged for LLIN on distribution day, and delivered educational messages on malaria and the importance of sleeping under a treated net. LLIN distribution was done at fixed sites at the ‘health area’ level and each household presented their coupon in exchange for LLINs. The number of LLINs to be allocated per household was calculated according to household size as follows: 1–2 persons = 1 LLIN; 3–5 persons = 2 LLINs; 6–8 persons = 3 LLINs; 9 and more persons = 4 LLINs.

#### Door-to-door (hang up) strategy

This strategy was used to distribute nets in 9 of the 44 HZ in Kasaï Occidental Province. Teams of 3 to 4 community volunteers visited each household sequentially at the moment of distribution. They were responsible for household registration (recording number of people, sleeping spaces, nets, etc.), giving nets and hanging them with the head of the household or another household member. The household registration and the delivery/hanging of nets were conducted in one visit. Community volunteers were provided hammers, string and nails for this purpose. Contrary to the fixed strategy, the number of LLIN per household here was calculated based on the number of sleeping spaces, with a ratio of one LLIN per sleeping space. Community volunteers were also trained in the use of smartphones to collect household data (socio-demographic, health-seeking behaviour, use of malaria prevention measures, etc.) and delivered educational messages about malaria and the importance of net use.

### Study design and sample size

A cross-sectional household based survey was conducted 2 months before and repeated 6 months after the mass LLIN distribution campaign. The pre-campaign survey took place in October 2014 and the post-campaign survey was conducted in July 2015. Sample size calculation was based on LLIN coverage of 55% before the campaign (Kinshasa School of Public Health 2012, unpublished report) and 85% after the campaign, a precision of 5 and 80% power. The resulting number of HZ to be sampled was calculated as 10 for the pre-campaign survey and 22 for the post-campaign survey (of which the 10 HZ from the pre-campaign survey were kept). In both surveys, 51 households were sampled per HZ.

A multi-stage cluster sampling method was used to select households. Health Zones were randomly selected from a complete list. To ensure sufficient representation from the door-to-door strategy (conducted in 9 of the 44 HZ), 2 of the 10 pre-campaign HZ and 5 of the 22 post-campaign HZ were randomly selected from those nine that received the door-to-door strategy. In each selected HZ, three HA were randomly selected from a complete list. In each HA, an exhaustive list of streets (for urban areas) and villages (for rural areas) with their corresponding populations was drawn up and three streets or villages were randomly selected from this list. A total of 17 households were sampled in each HA (to give a total of 51 households per HZ) and the number of households to be surveyed in each of the three selected villages/streets from the HA was proportional to the size of the street or village. Households were identified by systematic random sampling. A total of 509 households were surveyed in the pre-campaign and 1121 in the post-campaign.

### Data collection

#### Household survey questionnaire

In all selected households the head or another responsible member of the household was interviewed after written informed consent was obtained. Interviewees were asked questions on all household members (sex, education level, occupation, whether they slept under net previous night), on all nets in the household (type, source, location and if it was slept under the previous night) as well as general information about the house including number of sleeping spaces and malaria knowledge. LLIN ownership and use were established by respondent self-report, however data collectors also requested to observe all nets available in the household at the time of the visit. The survey teams recorded the presence of material goods in the household such as radios, electricity and various types of livestock, and also noted types of toilets, types of roof and wall construction. From this, a composite household wealth index was created using a principal components analysis (PCA) to determine households’ socioeconomic status [18]. Longitude and latitude coordinates of all surveyed households were recorded on-site using the integrated global positioning system (GPS) of the data collection devices. Data were collected using a standardized questionnaire electronically programmed on tablets (Samsung Table 3) running Google Android operating system and equipped with Open Data Kit software (ODK, University of Washington & Google Foundation). This questionnaire was adapted from the standard Malaria Indicator Survey household questionnaire from the Roll Back Malaria (RBM) partnership [19]. It was developed in French with oral translation into local language and dialects, and pre-tested prior to use in the field. After daily quality control checks by field supervisors, completed data were sent regularly to the central server housed at the Swiss Tropical and Public Health Institute (Swiss TPH) for distant access and verification by members of the coordination team.

#### Blood testing

During the post-survey only, all eligible children aged 6–59 months present in surveyed households were tested for malaria using the SD Bioline three bands *Plasmodium falciparum/*Pan malaria Rapid Diagnostic Test (RDT) (Standard Diagnostics, Kyonggi, Republic of Korea) and had haemoglobin levels measured using a blood haemoglobin photometer (HemoCueHb201 + Ängelholm, Sweden). Children with positive malaria tests were given free treatment with an artemisinin-based combination therapy (ACT), in particular artesunate–amodiaquine (AS–AQ), the official first-line malaria treatment at the time of the survey in the DRC. For children with signs of complicated malaria or low haemoglobin levels, parents were advised to visit the nearest health facility.

#### Collection of cost data

A comparative financial cost analysis between the fixed delivery and door-to-door distribution strategies was carried out from the provider’s perspective, which was defined as the cost incurred by implementation agencies. All the distribution activities including LLIN procurement and delivery were conducted separately by the two implementation agencies. Cost components of each distribution strategy were identified using the ingredients approach. Costs were collected retrospectively using financial expenditure records to capture financial costs from the accountant service of the implementing agencies using a standardized spreadsheet developed by the NMCP. Costs related to research activities were excluded. The procurement cost of LLIN including purchase cost, shipment and custom clearance were included in the analysis. For the fixed delivery strategy, some of the costs were collected in Great British Pound (GBP) and converted into US Dollars (USD) applying the 2015-year of expenditure-average exchange rate of USD 15,283 to the GBP [20]. For the door-to-door strategy, costs were collected in USD. For each distribution strategy the delivery cost per LLIN (i.e. total cost per net delivered) was calculated. Calculations of ‘per LLIN’ costs under each distribution strategy were based on the total number of LLINs recorded as distributed per strategy. Costs are presented in 2015 USD.

#### Measurements and indicators’ definition

Standard malaria household survey indicators were measured as recommended by the RBM Monitoring and Evaluation Reference Group (MERG) [19] as follows: prevention indicators: (1) proportion of households with at least one LLIN; (2) proportion of households with at least one LLIN for every two people; (3) proportion of population with access to a LLIN within their household. This indicator estimates the proportion of the population that could potentially be covered by existing LLIN, assuming each LLIN can be used by two people within a household. The calculation used took into account those household members who actually slept under an LLIN the previous night considered as having access to a LLIN within the household. The indicator needs an intermediate variable which is “potential users” calculated by multiplying the number of LLIN in each household by two. The indicator is then calculated by dividing the sum of all potential and actual LLIN users in the sample by the total number of individuals who spent the previous night in surveyed households. Full details are described by Kilian et al. [21]); (4) proportion of population that slept under a LLIN the previous night; (5) proportion of children under 5 years old who slept under a LLIN the previous night; (6) proportion of pregnant women who slept under a LLIN the previous night; (7) proportion of existing LLINs used the previous night. Case management indicators: (8) proportion of children less than 5 years old with fever in the last 2 weeks who had a finger or heel stick; (9) proportion of children less than 5 years old with fever in the last 2 weeks for whom advice or treatment was sought; (10) proportion receiving an ACT (or other appropriate treatment), among children less than 5 years old with fever in the last 2 weeks who received any anti-malarial drugs. Morbidity indicators: (11) malaria prevalence, defined as the proportion of children aged 6–59 months with a positive RDT; (12) anaemia prevalence, defined as the proportion of children aged 6–59 months with haemoglobin rate <8 g/dl.

#### Data management and analysis

Data were extracted from the ODK aggregate server using the ODK Briefcase in the CSV format and imported into STATA version 13 (Stata Corporation College Station, TX, USA) for statistical analysis. Dichotomous outcomes were summarized as proportions with 95% confident intervals. Continuous outcomes were described using their mean and standard deviation, or median and 90% central range if the distribution was skewed. The Pearson Chi square was used to compare proportions. Bivariate associations between the primary outcome and hypothesized explanatory variables were first done to guide subsequent model building; odds ratios and 95% confidence intervals were produced using logistic regression. After testing individual bivariate associations, a backward selection procedure was used to create an optimal multivariate model while adjusting for potential confounders. To take into account clustering by HZ and HA, a multi-level mixed effects logistic regression model was used to assess the association between the outcome and explanatory variables. Clustering at street/village level was not accounted for in the analysis; clustering by HZ and HA explains most of the variability in the sample. Results are presented as adjusted odds ratios with their 95% confidence intervals.

## Results

### Households characteristics

Table 1 displays the characteristics of all surveyed households. During the pre-campaign survey, a total of 509 households were visited across 10 HZ including 3227 people of which 51.5% were female. The median (90% central range) number of persons per household was 6 (2–12); the median number of children less than 5 years of age per household was 1 (0–3). In the post-distribution survey, 1121 households were sampled of which 868 were from HZ that received LLIN through the fixed delivery strategy and 253 were from HZ that received LLIN through the door-to-door strategy. In total, 6157 people lived in the households surveyed, 4886 in HZ with fixed strategy and 1271 in HZ with door-to-door strategy and in both strategies, about half (50.5%) of the survey population were female (fixed: 50%; door-to-door: 52.5%). The median number of persons per household was 5 (2–10) [fixed: 5 (2–10); door-to-door: 5 (2–9)] and the median number of children less than 5 years of age per household was 1 (0–3) [fixed: 1 (0–3); door-to-door: 1 (0–2)].

**Table 1:** Characteristics of surveyed households

Characteristics	Survey		Post survey by delivery strategy	
	Pre	Post	Fixed	Door-to-door
Number of households	509	1121	868	253
Number of individuals in sampled households	3227	6157	4886	1271
Percent female	51.5	50.5	50.0	52.5
Median (90% central range) number of people per household	6 (2–12)	5 (2–10)	5 (2–10)	5 (2–9)
Median (90% central range) number of children under 5 per household	1 (0–3)	1 (0–3)	1 (0–3)	1 (0–2)
Median (90% central range) number of nets per household	0 (0–2)	2 (0–4)	2 (2–4)	2 (2–4)

### Households’ LLIN ownership and intra household access to LLIN

Table 2 shows key malaria household indicators before and after the campaign. Table 3 shows post-distribution indicators by distribution strategy. The proportion of households owning at least one LLIN increased from 39.4% [95% CI 32.2–47.0%] before the distribution to 91.4% [95% CI 88.8–93.4%] after the distribution (Table 2). Household ownership of at least one LLIN after the distribution was significantly higher in HZ with fixed delivery strategy compared to those with door-to-door strategy with a mean of 92.5% [95% CI 90.2–94.4%] versus 85.2% [95% CI 78.5–90.0%], respectively (*χ2* = 5.71 *p* = 0.026) (Table 3).

**Table 2:** Key malaria household survey indicators before and after the mass distribution campaign

Indicators	Pre (% CI)	Post (% CI)	p value
Proportion of households with at least one LLIN	39.4 [32.2–47.0]	91.4 [88.8–93.4]	<0.001
Proportion of households with at least one LLIN for every two people	4.1 [2.5–6.5]	41.1 [36.1–46.2]	<0.001
Proportion of population with access to an LLIN in their household	22.2 [17.9–27.3]	80.7 [76.8–84.6]	<0.001
Proportion of the population that slept under an LLIN the previous night	18.0 [14.5–22.2]	68.3 [62.9–73.3]	<0.001
Proportion of children <5 years who slept under an LLIN the previous night	23.8 [18.0–30.6]	73.7 [67.8–78.9]	<0.001
Proportion of pregnant women who slept under an LLIN the previous night	20.9 [12.7–32.4]	74.0 [63.9–82.2]	<0.001
Proportion of existing LLINs used the previous night	82.2 [75.9–87.2]	66.7 [61.5–71.5]	<0.001
Proportion of children <5 years with fever in the last 2 weeks		37.7 [29.5–46.0]	
Proportion of children <5 years with fever in last 2 weeks who had a finger or heel stick		26.1 [20.5–31.6]	
Proportion of children <5 years with fever in the last 2 weeks for whom advice or treatment was sought		31.0 [23.1–38.9]	
Proportion receiving an ACT (or other appropriate treatment), among children under five years old with fever in the last 2 weeks who received any anti-malarial drugs		32.6 [15.7–49.4]	
Proportion of children aged 6–59 months with malaria infection		44.8 [34.7–55.0]	
Proportion of children aged 6–59 months with a hemoglobin measurement of <8 g/dl		14.6 [11.0–18.3]

**Table 3:** Key malaria household survey indicators by distribution strategy

Indicators	Fixed (% CI)	Door-to-door (% CI)	*χ2*	p value
Proportion of households with at least one LLIN	92.5 [90.2–94.4]	85.2 [78.5–90.0]	5.71	0.026
Proportion of households with at least one LLIN for every two people	44.1 [38.7–49.7]	30.9 [22.7–40.6]	5.14	0.034
Proportion of population with access to an LLIN in their household	85.0 [81.1–88.2]	75.8 [65.3–83.9]	2.45	0.131
Proportion of the population that slept under an LLIN the previous night	69.6 [63.1–75.5]	65.7 [52.7–76.7]	0.07	0.791
Proportion of children under 5 years old who slept under an LLIN the previous night	74.8 [67.9–80.7]	71.6 [57.2–82.6]	0.12	0.729
Proportion of pregnant women who slept under an LLIN the previous night	79.6 [64.0–89.6]	65.0 [34.4–86.9]	1.08	0.310
Proportion of existing LLINs used the previous night	63.7 [58.3–68.8]	76.9 [68.0–83.9]	9.01	0.007
Proportion of children aged 6–59 months with malaria	37.8 [25.9–51.5]	64.9 [39.6–83.9]	2.78	0.110
Proportion of children aged 6–59 months with a hemoglobin measurement of <8 g/dl	13.4 [10.1–17.6]	11.6 [6.6–19.6]	0.29	0.597

LLIN universal coverage, measured as the proportion of households with at least one LLIN for every two people increased from 4.1% [95% CI 2.5–6.5%] in the pre-campaign to 41.1% [95% CI 36.1–46.2%] in the post-campaign (Table 2). After the distribution, the proportion of households owning at least one LLIN for every two people was significantly higher in HZ with fixed delivery strategy compared to HZ with door-to-door strategy with a mean of 44.1% [95% CI 38.7–49.7%] versus 30.9% [95% CI 22.7–40.6%], respectively (*χ2* = 5.14 *p* = 0.034) (Table 3). The average number of LLIN in the surveyed households was approximately one for every 2.5 people (Fixed:1 LLIN:2.4 people; door-to-door: 1 LLIN:3 people).

To assess the performance of each delivery strategy, the proportion of households reached during the campaign (proportion of households with at least 1 LLIN from the campaign) was calculated while the proportion of households with sufficient LLIN (1 LLIN for every two people) was calculated among those households that received at least 1 LLIN from the campaign to assess the efficiency of each allocation method. The proportion of households with at least 1 LLIN from the campaign (households reached) was significantly higher in HZ that received LLIN through fixed delivery strategy compared to those that received LLIN through the door-to-door strategy with a mean of 91.4% [95% CI 89.1–93.7%] versus 79.0% [95% CI 70.2–87.8%], respectively (*χ2* = 13.87 *p* < 0.001). Among households reached, the proportion of those that received enough LLIN (1 LLIN for 2 people) did not significantly vary by net allocation method (net per person: 50.0% [95% CI 45.6–54.5%]; net per sleeping space: 42.7% [95% CI 29.2–56.2%]; *χ2* = 1.90 *p* = 0.186).

**Fig. 2:**
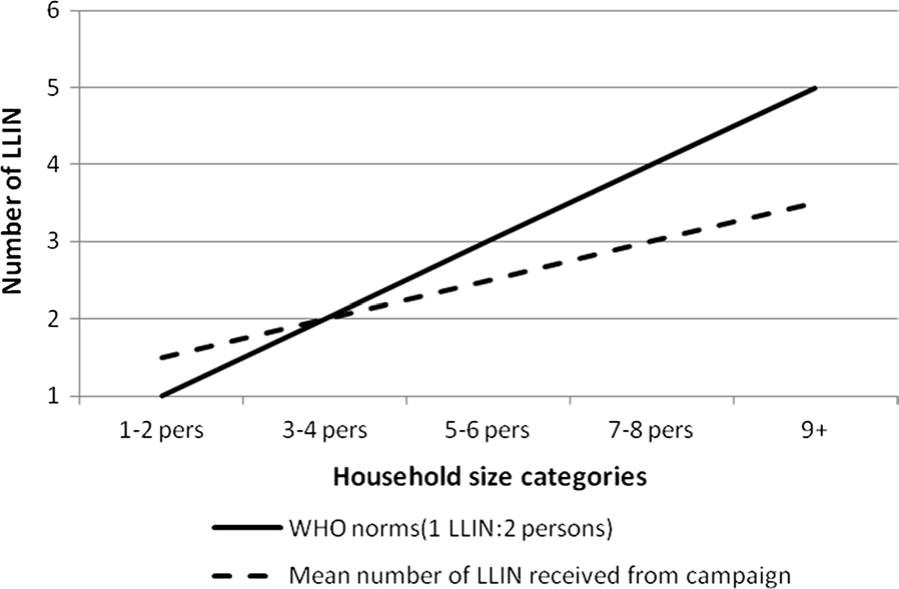
Number of LLIN received from the mass distribution campaign by household size

In households containing more than four people, regardless of the delivery strategy, the mean number of LLIN received from the campaign was consistently lower than the WHO recommendation of one LLIN for every two people (Fig. 2). Population access to LLIN within the household increased from 22.2% [95% CI 17.9–27.3%] pre-campaign to 80.7% [95% CI 76.8–84.6%] post campaign (Table 2). The post distribution access to a LLIN within the household did not vary by distribution strategy (fixed: 85.0% [95% CI 81.1–88.2%]; door-to-door: 75.8% [95% CI 65.3–83.9%]; *χ2* = 2.45 *p* = 0.131) (Table 3).

### LLIN use

Overall LLIN use increased from 18.0% [95% CI 14.5–22.2%] in the pre-distribution survey to 68.3% [95% CI 62.9–73.3%] after distribution. The overall use of LLIN was not statistically different between HZ with different distribution strategies (fixed: 69.60% [95% CI 63.1–75.5%]; door-to-door: 65.7% [95% CI 52.7–76.7%]; *χ2* = 0.07 *p* = 0.791) (Table 3).

**Fig. 3:**
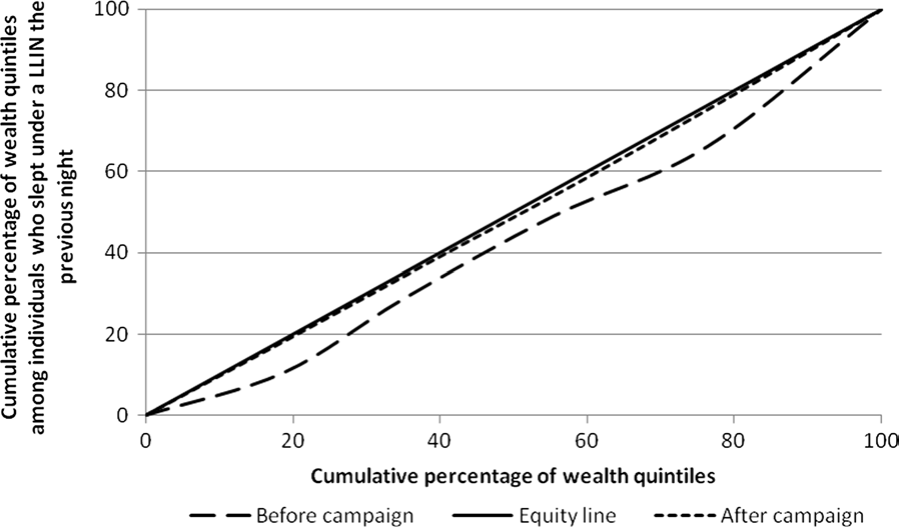
Lorenz concentration curve showing equity in LLIN use before and after the campaign. Concentration index (95% CI). Before campaign: 0.12 (0.02–0.22). After campaign: 0.02 (0.00–0.02)

Before the mass distribution campaign, LLIN use was lowest among the poorest wealth quintile and progressively increased with increasing wealth with a concentration index of 0.12 [95% CI 0.02–0.22]. After the distribution no specific pattern was observed in the LLIN use with regard to the socio economic status of the household with a concentration index of 0.02 [95% CI 0.00–0.02]. Figure 3 presents the Lorenz concentration curve describing the equity in LLIN use before and after the campaign.

**Fig. 4:**
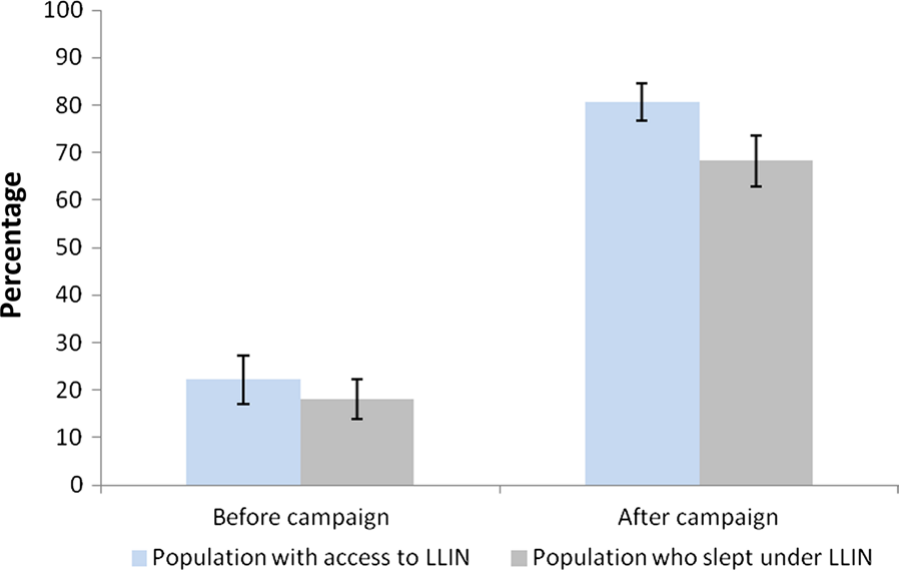
Population access and use before and after the mass distribution campaign

After the mass distribution, LLIN use was significantly higher in households with universal coverage (1 LLIN for 2 people) with a mean of 82.0% [95% CI 76.6–87.4%] versus 58.4% [95% CI 52.2–64.6%] (*χ2* = 44.70 *p* < 0.001). During both pre- and post-distribution surveys, at least 80% (pre: 81.1%; post: 84.6%) of the population with access to a LLIN within their household slept under it the previous night (Fig. 4).

Approximately one quarter (23.8%) of children less than 5 years of age slept under a LLIN before the distribution while there were three quarters (73.7%) after the distribution (Table 2). The post-distribution use of LLIN by children less than 5 years of age did not vary by distribution strategy (fixed: 74.8% [95% CI 67.9–80.7%]; door-to-door: 71.6% [95% CI 57.2–82.6%]) (Table 3).

**Fig. 5:**
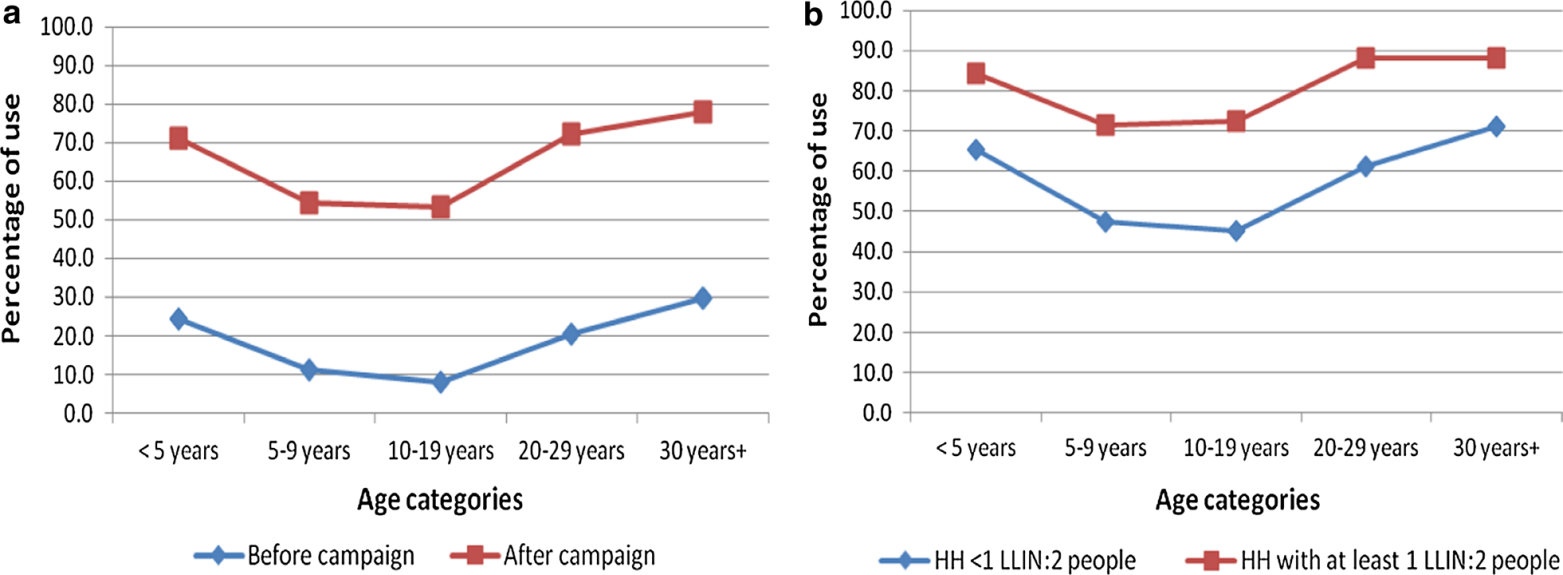
Age-specific use of LLIN. Before and after the mass distribution campaign (**a**). By coverage level after the mass distribution campaign (**b**)

In both pre- and post-distribution surveys, the use of LLIN varied strongly across different age groups, with the lowest use rate observed in the age group of 5–19 years old (Fig. 5a). Even in households with universal coverage (1 LLIN for 2 people), age specific use of LLIN consistently showed the same pattern (Fig. 5b).

Use of LLIN by pregnant women increased from 20.9% [95% CI 12.7–32.4%] to 74.0% [95% CI 63.9–82.2%] before and after the distribution respectively (Table 2). The latter did not vary by distribution strategy (fixed: 79.6% [95% CI 64.0–89.6%]; door-to-door: 65.0% [95% CI 34.4–86.9%]) (Table 3).

After the distribution campaign, on average 66.7% [95% CI 61.5–71.5%] of existing LLIN were used the previous night. This proportion was slightly higher in HZ with door-to-door strategy compared to those with fixed strategy with a mean of 76.9% [95% CI 68.0–83.9%] versus 63.7% [95% CI 58.3–68.8%] (*χ2* = 9.01 *p* = 0.007) (Table 3). On average, 2.4 sleepers shared the same LLIN the previous night. Overall, around 60% of existing LLIN used the previous night had one or two sleepers, considered as appropriate coverage while the rest had more than two sleepers.

During the post-distribution survey, about 60% of interviewed household members reported to have heard or seen a message on malaria or LLIN in the last 30 days. The most commonly mentioned sources of messages were community health workers (46.2%), health centres (33.7%) and radio (32.3%), TV and other mass media channels were mentioned by about 10% of respondents. The most commonly recalled message content were “nets prevent malaria” (66.6%) and “use a net every night” (67.6%).

### LLIN characteristics

During the post-distribution survey, a total of 2479 LLINs were recorded in surveyed households; 2121 (85.6%) of which were observed. Of the 2121 LLIN observed, 70.6% [95% CI 64.7–76.4%] were hung at the time of the interview. The proportion of LLIN hung per strategy was significantly higher in HZ with door-to-door strategy compared to the fixed delivery strategy with a mean of 90.1% [95% CI 86.0–94.2%] versus 67.5% [95% CI 61.6–73.3%] respectively (*χ2* = 8.56 *p* = 0.008). Nearly all (98%) of the LLINs observed in households during the post-distribution survey were marked Permanet® and were obtained from the mass distribution campaign.

Overall, 60% of households reported to have hung their LLINs the same day or the day following its reception but this proportion was higher in HZ with door-to-door strategy than in HZ with fixed delivery strategy (90.1 versus 52.6%). In HZ with fixed strategy, nearly all households (98.7%) reported their LLINs were hung by a household member, whereas in HZ with door-to-door strategy, over half of the households (56.5%) reported their LLINs were hung by a member of the distribution team and 43.5% by a household member. Nearly all households (97.7%) encountered no problems hanging their LLIN in both strategies.

### Health-seeking behaviour and malaria morbidity

Data on health-seeking behaviour and malaria morbidity were collected only during the post-distribution survey. More than one-third (37.7% [95% CI 29.5–46.0%]) of children less than 5 years old had fever in the 2 weeks preceding the survey. Advice or treatment was sought for 31.0% [95% CI 23.1–38.9%] of them and a quarter (26.1%; [95% CI 20.5–31.6%]) had a finger or heel prick. Among these children less than 5 years of age who had fever in the 2 weeks before the survey and who received any anti-malarials, 32.6% [95% CI 15.7–49.4%] received ACT (Table 2).

Malaria prevalence among children less than 5 years old was 44.8% (95% CI 34.7–55.0%) and the proportion of children aged 6–59 months with a haemoglobin measurement of <8 g/dl was 37.7% [95% CI 29.5–46.0%] (Table 2). Malaria and anaemia prevalence was not significantly different between distribution strategies (Table 3).

### Determinants of LLIN use

The contribution of different factors associated with LLIN use before and after the distribution is shown in Tables 4 and 5. During the pre-distribution survey, there was no evidence of association between use of LLIN and gender, while significant heterogeneities were observed in LLIN use among age groups. Compared to children less than 5 years of age, individuals aged 5–19 years were significantly less likely to sleep under a LLIN (OR = 0.26 [95% CI 0.19, 0.34]) and those aged 30 years and above were significantly more likely to use a LLIN (OR = 1.40 [95% CI 1.06, 1.86]). A higher educational level of the head of the household was associated with increased odds of sleeping under a LLIN (OR = 2.67 [95% CI 1.15, 6.19]). Individuals living in households whose head was employed were also significantly more likely to use a LLIN than those of other occupations (OR = 1.81 [95% CI 1.06, 3.09]). There was no evidence of an association between LLIN use and the number of persons per sleeping space, the knowledge of malaria transmission or the exposition to a sensitisation message on malaria/LLIN. The least poor socio-economic quintile (compared with the poorest) was associated with significant increased odds of sleeping under a LLIN (OR = 2.79 [95% CI 1.54, 5.07]).

**Table 4:** Logistic regression model showing determinants of LLIN use before the mass distribution campaign

Variable	*n*	(%)	Univariate analysis			Multivariate analysis		
			OR	95% CI	*p* value	OR	95% CI	*p* value
Sex								
Male	1413	17.7	1			1		
Female	1582	19.1	1.17	0.96–1.43	0.118	1.15	0.93–1.42	0.190
Age								
<5 years	576	24.3	1			1		
5–19 years	1328	9.3	0.26	0.19–0.35		0.26	0.19–0.34	
20–29 years	383	20.6	0.73	0.52–1.02		0.80	0.56–1.13	
≥30 years	708	29.5	1.2	0.92–1.57	<0.001	1.40	1.06–1.86	<0.001
Education of the head of the household								
No education	73	15.1	1			1		
Primary	640	11.3	1.06	0.50–2.22		1.20	0.55–2.63	
Secondary	2066	18.2	1.8	0.89–3.64		1.59	0.74–3.42	
Superior and above	216	43.1	3.8	1.78–8.13	<0.001	2.67	1.15–6.19	0.010
Occupation of the head of the household								
Without occupation	187	13.4	1			1		
Farmer	1160	12.4	0.87	0.53–1.42		0.83	0.49–1.41	
Merchant	927	15.3	1.14	0.70–1.85		0.93	0.54–1.60	
Employed	721	33.4	2.42	1.51–3.90	<0.001	1.81	1.06–3.09	<0.001
Persons per sleeping space								
2 or less	1752	19.18	1			1		
More than 2	1243	17.38	0.79	0.64–0.97	0.025	1.04	0.58–1.88	0.889
Wealth quintile								
Poorest	558	10.6	1			1		
Second	496	20.4	2.67	1.78–4.00		2.38	1.54–3.68	
Middle	624	17.8	2.54	1.66–3.88		2.23	1.40–3.54	
Fourth	637	15.2	1.93	1.23–3.02		1.82	1.06–3.11	
Least poor	680	27.1	3.23	2.00–5.23	<0.001	2.79	1.54–5.07	<0.001
Knowledge transmission								
No	775	13.7	1			1		
Yes	2220	20.1	1.29	0.98–1.29	0.064	1.20	0.89–1.60	0.226
Heard a message on malaria/ITN last month								
No	1113	16.4	1			1		
Yes	1882	19.6	1.14	0.90–1.45	0.274	0.97	0.74–1.26	0.798

**Table 5:** Logistic regression showing determinants of LLIN use after the mass distribution campaign

Variable	*n*	(%)	Post distribution					
			Univariate analysis			Multivariate analysis		
			OR	95% CI	*p* value	AOR	95% CI	*p* value
Sex								
Male	2746	66.4	1			1		
Female	2913	67.2	1.05	0.93–1.18	0.458	1.05	0.93–1.20	0.422
Age								
<5 years	1308	71.6	1			1		
5–19 years	2164	54.1	0.41	0.35–0.49		0.39	0.33–0.46	
20–29 years	706	72.5	1.03	0.83–1.28		0.97	0.77–1.23	
≥30 years	1481	78.4	1.49	1.24–1.79	<0.001	1.46	1.21–1.78	<0.001
Education of the head of the household								
No education	397	58.2	1			1		
Primary	1599	62	1.35	1.04–1.74		1.28	0.97–1.69	
Secondary	3265	68.8	2.08	1.63–2.66		1.92	1.46–2.52	
Superior and above	398	78.1	2.95	2.06–4.23	<0.001	2.29	1.52–3.45	<0.001
Occupation of the head of the household								
Without occupation	355	63.9	1			1		
Farmer	2748	63.8	0.91	0.70–1.19		1.40	0.94–2.09	
Merchant	1397	64.3	1.06	0.81–1.39		1.62	0.94–2.79	
Employed	1159	77.8	1.95	1.47–2.59	<0.001	3.73	1.75–8.38	<0.001
Persons per sleeping space								
2 or less	3722	70.0	1			1		
More than 2	1937	65.2	0.84	0.74–0.96	0.010	0.97	0.66–1.41	0.862
Distribution strategy								
Fixed	4577	67.2	1			1		
Door-to-door	1082	65.3	0.87	0.47–1.61	0.655	0.80	0.40–1.62	0.538
Wealth quintile								
Poorest	1114	63.6	1			1		
Second	1081	66.2	1.04	0.84–1.27		0.94	0.71–1.25	
Middle	1137	64.6	1.47	1.14–1.88		1.51	0.98–2.33	
Fourth	1105	68.3	1.72	1.33–2.23		1.84	0.98–3.37	
Least poor	1222	70.8	1.49	1.12–2.00	<0.001	1.53	0.67–3.46	0.061
Knowledge transmission								
No	1121	62.1	1			1		
Yes	4538	68.0	1.47	1.25–1.73	<0.001	1.39	1.16–1.68	<0.001
Heard a message on malaria/ITN last month								
No	2110	61.4	1			1		
Yes	3549	70.0	1.74	1.51–2.00	<0.001	1.57	1.34–1.84	<0.001
At least 1 LLIN/2 people								
No	3730	58.8	1			1		
Yes	1929	82.3	3.35	2.89–3.88		3.79	3.21–4.49	<0.001

Following the mass distribution, no association was found between gender and the use of LLIN as before. The age specific use of LLIN showed the same pattern as before the distribution, with the 5–19 years olds having the lowest odds of LLIN use (OR = 0.39 [95% CI 0.33, 0.46]) and the 30 years and above being more likely to use a LLIN (OR = 1.46 [95% CI 1.21, 1.78]) compared with children less than 5 years. As before the distribution, occupation and educational level of the head of the household were significantly associated with the use of LLIN. There was no evidence of association between the use of LLIN and the distribution strategy. Individuals living in households whose head knew the cause of malaria (OR = 1.39 [95% CI 1.16, 1.68]) or have heard about malaria or LLIN in the last month (OR = 1.57 [95% CI 1.34, 1.84]) were more likely to sleep under a LLIN. The socio-economic status of the household was not associated with LLIN use. Individuals living in households owning at least one LLIN for every two people had the highest odds of sleeping under a LLIN (OR = 3.79 [95% CI 3.21, 4.49]).

### Cost analysis

Costing details for both strategies are shown in Table 6. The total financial cost of the campaign from the provider perspective was USD 22.84 million (USD 18.71 million for the fixed delivery strategy and USD 4.13 million for the door-to-door strategy). The total financial cost per LLIN distributed was USD 6.59 (USD 6.58 for the fixed distribution strategy and USD 6.61 for the door-to-door strategy) of which USD 4.08 were used for LLIN purchase and custom clearance and USD 2.51 were for LLIN transport, storage, training, mobilisation/IEC, management and M&E. Overall, LLIN cost, transport and storage comprise around 80% (87.3% for the fixed delivery strategy and 70.3% for the door-to-door strategy) of the total financial cost. The cost of LLIN purchase was higher for the fixed strategy compared to the door-to-door strategy (USD 4.17 versus USD 3.66) while the non-LLIN costs were lower for the fixed strategy compared to the door-to-door strategy (USD 2.41 versus USD 2.95).

**Table 6:** Financial costs of the LLIN distribution by cost category and delivery strategy

	Door-to-door	Fixed	Combined						
Number of LLIN distributed	624,532	2,843,442	3,467,974						
Total financial cost (2015 USD)	4,130,050	18,706,824	22,836,874						
Financial cost per LLIN delivered	6.61	6.58	6.59						

Cost of LLIN Campaign (2015 USD) per category	Cost	Cost per LLIN	% of cost	Cost	Cost per LLIN	% of cost	Cost	Cost per LLIN	% of cost
LLINs	2,287,500	3.66	55.4	11,858,176	4.17	63.4	14,145,676	4.08	61.9
Transport and storage	613,920	0.98	14.9	4,477,243	1.57	23.9	5,091,163	1.47	22.3
Personnel	567,484	0.91	13.7	555,023	0.20	3	1,122,507	0.32	4.9
Trainings	140,997	0.23	3.4	660,994	0.23	3.5	801,991	0.23	3.5
Office, supplies and equipment	438,654	0.70	10.6	566,167	0.20	3	1,004,821	0.29	4.4
IEC	20,995	0.03	0.5	469,300	0.17	2.5	490,295	0.14	2.1
M&E	60,500	0.10	1.5	119,921	0.04	0.6	180,421	0.05	0.8

## Discussion

Concerted efforts to scale up LLIN coverage through a free mass distribution campaign in the Kasaï Occidental province have rapidly increased ownership and use of LLIN. In terms of coverage, RBM targets of 80% of households owning at least one LLIN and 80% of population having access within their household have been achieved. Universal coverage (defined as households with at least one LLIN for every two people) though below the 80% target, has shown a remarkable tenfold increase from 4 to 41%. These findings are consistent with what is known about the effectiveness of mass distribution campaigns to quickly scale-up LLIN coverage in low coverage areas [5–7, 22]. However, considering there had been a previous mass distribution campaign in 2011 with high coverage values, the ownership and use indicators found in the pre-distribution survey were surprisingly low.

Following a universal free mass distribution campaign, the fact that less than half of surveyed households had at least one LLIN for every two people can be surprising. This highlights a limitation of the distribution campaign in quantifying the number of LLIN allocated per household, in particular for households of more than four members. A study conducted in Sierra Leone 6 months after a mass distribution campaign also showed that when limiting the maximum number of LLIN one household can receive, households with more than five residents were less likely to have sufficient LLIN to cover all occupants [5].

Despite a dramatic increase in LLIN access and use overall, significant heterogeneities were observed in LLIN use among age groups, with the lowest use rate observed in the age group of 5–19 years old. The age specific pattern observed has been reported by other researchers in different contexts including DRC, [11, 23–25]. Interestingly, in this study, the same pattern was observed even in households possessing sufficient numbers of LLIN to cover all residents, suggesting a behavioural gap in LLIN use among older children and adolescents. The lower LLIN use rate obviously put this age group at higher risk of malaria prevalence as reported in other studies [26, 27].

Findings from this study also showed that both before and after the campaign, at least 80% of those with access to a LLIN used it the previous night. While remarkable efforts are made to increase access to LLIN, it is also important that the NMCP focus on developing behaviour change communications strategy and plan to promote LLIN use in the general population as well as in specific group such as older children and adolescents.

Contrary to what could be expected, results of this study showed that the fixed delivery strategy reached a much higher proportion of households compared to the door-to-door strategy with 91.4% of households with at least 1 LLIN from the campaign versus 79.0% respectively. However, among those households reached by either strategy, the net allocation method (which differed by strategy) did not influence whether a household had sufficient LLIN for one per two people. A multi country comparison of LLIN delivery strategies based on 14 surveys from five African countries did not find a significant association between delivery strategy and ownership of a net from the campaign but found a positive association between sleeping space allocation and enough LLIN in the household [28].

Only half of surveyed households in areas where the hang up approach was implemented reported their LLIN was hung by a member of the distribution team. However, of those that were hung by a member of the distribution team, a higher proportion were still hung and used the previous night compared to those not hung by a member of the distribution team as also noted by other researchers [5, 10]. However, this did not necessarily result in higher LLIN use rates among the population, indicating that the distribution strategy has no influence on LLIN use. A cluster randomized controlled trial conducted in Uganda showed that additional hang up activities following a mass distribution campaign did not provide any additional impact on net use [29]. In this study, the strongest determinant of LLIN use—having sufficient LLIN to cover all households’ residents—did not differ significantly by distribution strategy.

As could be expected after a free LLIN mass distribution campaign that targeted the entire population at risk for malaria, equity in household LLIN coverage and individual use of LLIN has been improved as demonstrated by the Lorenz curve meeting the equity line as well as the concentration index shifting from positive to close to zero values. These findings corroborate results from other mass distribution campaigns showing equitable LLIN ownership and use [8, 22, 30, 31].

Despite higher coverage and reported use of LLIN 6 months after a free mass distribution of LLIN, malaria prevalence among under-fives remains high in the province. The overall malaria prevalence among children aged 6–59 months found in this study was higher than the national average of 31% prevalence reported by the DHS [17]. This high malaria prevalence rate calls for further investigation of possible contributors. As an attempt to identify factors explaining high malaria rates in northern Ghana, Monroe et al. found that under-usage of LLINs at times when they could confer maximum protection as well as a variety of outdoor night-time activities, including outdoor sleeping were factors that could have potentially contributed to high rates of malaria in that setting [32]. In this study, the prevalence of anaemia was high and consistent with findings of other researchers [26], however additional factors common in this setting such as malnutrition [17] and sickle cell anaemia [33] play a role in the occurrence of this condition.

Access to diagnostic testing and malaria treatment is very low; efforts should be made to increase availability of RDT and ACT in both public and private sectors. To estimate the cost of implementation, a comparative financial cost analysis providing the cost per LLIN delivered was more suitable than a cost effectiveness analysis. For both fixed delivery distribution and door-to-door strategies, the average cost per LLIN distributed was consistent with findings of other researchers [34]. As expected, the highest proportion of cost was attributable to the purchase cost of the LLIN. Compared to the fixed strategy, the average cost per LLIN distributed was slightly higher in the door-to-door strategy with the personnel cost being the second highest single cost position after LLINs. This is consistent with the additional cost associated with hang up activities as reported by other researchers [29, 35]. While the overall non-LLIN cost was lower for the fixed delivery strategy, the costs of transportation and storage were higher for the fixed delivery strategy compared to the door-to-door strategy. The fact that the 35 HZ with fixed delivery strategy were spread over 4 districts whereas the 9 HZ with door-to-door strategy are all in 1 district might have resulted in higher logistics costs in fixed delivery strategy.

This study has limitations. Although interviewers were required to observe LLINs owned by households, most net results reported in this study relies on data reported by respondents, thus they are prone to recall and information bias. LLIN may be more subject to over-reporting due to social desirability bias. As RDTs were used for malaria diagnostic and parasite antigens (detected by the test) often persist up to 2 weeks post-treatment, some children previously treated for malaria might have tested positive within 14 days after treatment.

## Conclusions

This study demonstrates substantial improvements in LLIN coverage, use and equity. Although all RBM targets were not met, much progress has been made. In addition to antenatal and vaccination clinic programmes, other LLIN distribution strategies should be explored as part of a keep-up strategy in order to maintain high and equitable coverage over time. The very low ownership and use levels observed before the campaign in this study despite a previous mass distribution campaign in 2011 is a stark reminder of the need for a keep-up mechanism.

These results also suggest a revision of distribution guidelines especially with regard to LLIN quantification to better cover larger households and those not reached by the mass distribution campaign. Having sufficient numbers of LLIN to cover all residents in the household was the strongest determinant of LLIN use. As access to LLIN is increasing, results of this study suggest that behaviour change strategies should focus on interpersonal interventions to promote LLIN use in the general population and specific groups such as older children and adolescents. In the context of the present study setting, a fixed delivery strategy seems to be a better LLIN delivery option, as it was shown to be associated with higher levels of LLIN coverage and use indicators as well as lower delivery cost.
